# Effects of hormones on intestinal stem cells

**DOI:** 10.1186/s13287-023-03336-1

**Published:** 2023-04-26

**Authors:** Li Liu, Lilong Zhang, Chunlei Li, Zhendong Qiu, Tianrui Kuang, Zhongkai Wu, Wenhong Deng

**Affiliations:** Department of General Surgery, Renmin Hospital of Wuhan University, Wuhan, Hubei China

**Keywords:** Intestinal stem cells, Hormones, Stem cell, Therapy

## Abstract

The maintenance of intestinal renewal and repair mainly depends on intestinal stem cells (ISCs), which can also contribute to the growth of intestinal tumours. Hormones, which are vital signalling agents in the body, have various effects on the growth and replacement of intestinal stem cells. This review summarises recent progress in the identification of hormones associated with intestinal stem cells. Several hormones, including thyroid hormone, glucagon-like peptide-2, androgens, insulin, leptin, growth hormone, corticotropin-releasing hormone and progastrin, promote the development of intestinal stem cells. However, somatostatin and melatonin are two hormones that prevent the proliferation of intestinal stem cells. Therefore, new therapeutic targets for the diagnosis and treatment of intestinal illnesses can be identified by examining the impact of hormones on intestinal stem cells.

## Introduction

The intestine is an essential organ of the body and aids in digestion and selective nutrient absorption in addition to acting as a barrier against pathogenic intestinal microbes and releasing hormones [1]. Owing to intestinal motility and damage to the intestinal lumen, these protective and absorptive processes occur against a background of significant mechanical stress. As a result of extreme strain, intestinal epithelial cells continue to update at an exceptionally rapid pace, with the majority of mature cells living only for a few days [2]. Hormones in the body can affect this process and alter the number and function of intestinal. Intestinal stem cells (ISCs), which are responsible for maintaining the intestinal epithelium through constant renewal and proliferation of stem cells found in the intestinal crypts.

The largest endocrine organ in the body is the intestinal mucosa [3]. Intestinal hormones and associated peptides are found throughout the intestinal mucosa in endocrine cells, which can produce hormones in an autocrine or paracrine manner to affect cellular processes. As neurosecretory mediators of nerve impulses after nerve stimulation, intestinal hormones may also be released into the vasculature [4]. The dynamic homeostasis of intestinal epithelial cells depends on the balance between the capacity of ISCs to undergo self-renewal and differentiation. Evidence suggests that hormones control the ability of ISCs to differentiate through multiple pathways, such as endocrine metabolic pathways, while maintaining a balance between self-renewal and differentiation. Notably, differences between the small intestine (SI) epithelial stem cells and the large intestine (LI) epithelial stem cells have been identified. Because there is little retrograde cellular movement in the large intestine, the effective number of stem cells in the small intestinal crypts is twice that of the large intestinal crypts [5]. There are also differences in the regulation of small and large intestinal epithelial stem cells by some hormones. These differences may be used to explain why the prevalence of colorectal cancer is much higher than that of small bowel cancer. This review summarises recent studies on ISCs and hormones (Table 1) and highlights the effects of intestinal hormones on alterations in the intestinal status to control ISCs, which may offer new insights into the diagnosis and treatment of intestinal illnesses.

**Table 1:** Categorization of hormones associated with intestinal stem cells

Function	Hormones
Promoting ISCs proliferation	Thyroid hormones
	Glucagon-like peptide 2
	Androgens
	Insulin
	Leptin
	Growth hormones
	Corticotropin-releasing hormone
	Progastrin
Inhibiting ISCs proliferation	Somatostatin
	Melatonin
Associated with CSCs	Thyroid hormones
	Leptin
	Somatostatin
	Melatonin
	Progastrin

ISCs: Intestinal stem cells; CSCs: cancer stem cells

## Fundamental traits of intestinal stem cells

**Fig. 1:**
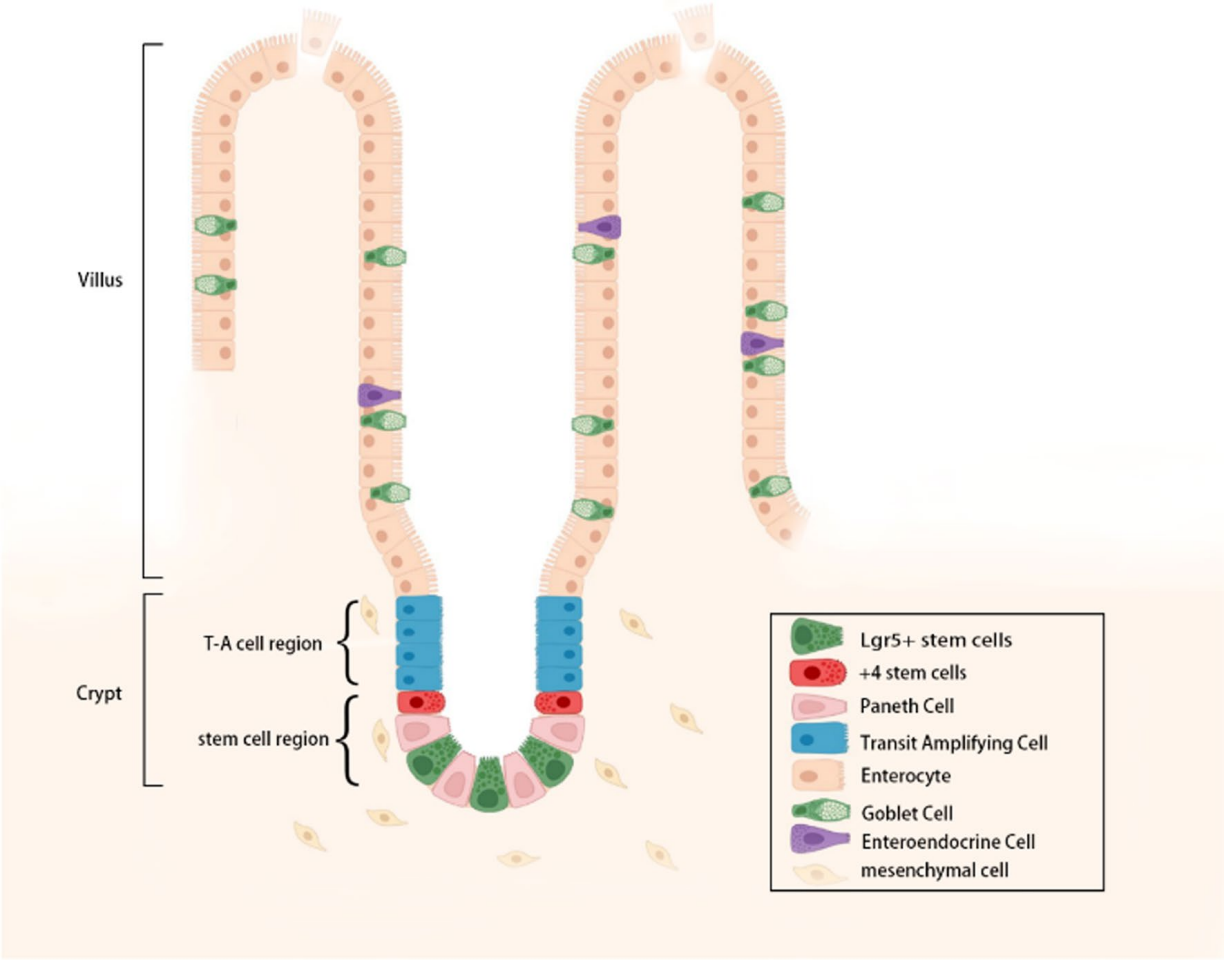
Diagram illustrating the arrangement of intestinal stem cells and the crypt–villus axis

The intestinal epithelium consists of a villi portion and a crypt portion. The villi protrude towards the intestinal lumen and play an important role in the digestion and absorption of food; the crypt is located between the villi in an invaginated form and is a proliferative area composed of intestinal stem cells and their progeny [6]. The mesenchymal microenvironment surrounding ISCs is known as the niche, which is a specialised and beneficial microenvironment in which stem cells reside. ISCs can rapidly undergo self-renewal and proliferation and are found near the base of the crypt. The niche offers all components necessary for the renewal, growth and function of ISCs and is crucial for maintaining stem cell signalling and renewal [7]. Transit-amplifying (TA) cells are created during the division of ISCs. Before gradually specialising and maturing into different types of intestinal cells, TA cells travel up the crypt–villus axis and divide 2–5 times. The cells die and shed from the terminals of the villi into the lumen to be removed [8] (Fig. 1). From the duodenum to the colon, where TA cells completely vanish, the length of the intestinal villi gradually decreases. The small intestine consists of villi that extend into the intestinal lumen and a crypt that inserts into the mucosa. The large intestine consists of the cecum and colon and has a crypt structure similar to that of the small intestine, but lacks the luminal-projecting villi [9]. ISCs can differentiate into one of the two major cell types: secretory cells, which mostly comprise Paneth, goblet and enteroendocrine cells, and absorptive cells, which include intestinal enterocytes [10]. Most intestinal stem cells divide to produce TA cells and then migrate upwards. Paneth cells are the exception. After differentiating and maturing from TA cells, they migrate downward instead of upward to the base of the crypt in an interphase distribution with stem cells. They can survive for 1–2 months at the base of the crypt and are subsequently removed by infiltrating macrophages after their death [11].

Leucine-rich repeat-containing G-protein-coupled receptor 5 (LGR5) and olfactomedin-4 (Olfm4) are the most characteristic ISC markers, which can help to distinguish between two types of stem cells, namely, circulating crypt base columnar (CBC) cells and quiescent ‘+4’ cells. To make up for the loss of Lgr5-labelled circulating CBC stem cells, the ‘+4’ locus, a largely dormant, injury-resistant stem cell class, multiplies after the intestine is exposed to radiation [12]. The other ISC markers include Musashi-1 (Msi1), B cell-specific Moloney murine leukaemia virus integration site 1 (Bmi1), ephrin receptor-B3 (EphB3), Sox9, c-myelocytomatosis (c-Myc), leucine-rich repeats and immunoglobulin-like domain protein 1 (Lrig1) [13–17].

The preservation, expansion and differentiation of ISCs are regulated by several important signalling pathways. ISCs have highly active WNT, BMP, Notch and EGF signalling pathways, which are important regulators of the maintenance, proliferation and differentiation of stem cells [18]. With increased signalling at the base of the crypt and decreased signalling in TA cell-rich regions, Wnt signalling in the small intestine promotes the self-renewal and proliferation of small intestinal stem cells. Contrary to Wnt signalling, the BMP signal is transduced in a gradient along the crypt–villus axis, with the expression of bone morphogenetic protein 2 (BMP2) and bone morphogenetic protein 4 (BMP4) ligands decreasing from top to bottom. BMP2 and BMP4 ligands are primarily released by mesenchymal cells in the villi and perivascular mesenchymal cells in the crypt. Almost all postnatal animals have active Notch signalling, which is a highly conserved signalling mechanism that mostly relies on cell–cell interactions to function. The fate of secretory and absorptive cells is mostly determined by Notch signalling, which suppresses the differentiation of secretory cells and promotes the differentiation of absorptive cells. EGF signalling promotes the growth of TA and intestinal stem cells in the crypt [19]. Other similar pathways include the JNK, JAK–STAT, Wg and insulin–IGF-1 signalling (IIS) pathways [20–23].

## Hormonal influence on intestinal stem cells

### Thyroid hormones

Thyroid hormones (triiodothyronine, T3) practically influence every biological process. T3 is crucial for the development of numerous adult organs and the maintenance of appropriate physiological functions in these organs. This is especially true during the maturation period of embryonic development, when plasma T3 levels are at their highest, which corresponds to the weeks and months before and after birth in mice and humans, respectively. Extreme developmental issues, such as intestinal maturation deficiencies, are driven by T3 deficits during embryonic development [24]. The hypothalamic–pituitary–thyroid axis controls the production and release of thyroid hormones through thyrotropin-releasing hormone (TRH) and thyroid-stimulating hormone (TSH), which are both produced by the thyroid gland [25]. Nuclear thyroid hormone receptors (TRs), which are found in all vertebrates, mediate the action of thyroid hormones on the genome. TRs are nuclear hormone receptor superfamily transcription factors. T3 response elements (TREs) control gene transcription by recruiting various cofactors in a T3-dependent manner, and TRs primarily bind to 9-cis-retinoic acid receptors (RXRs) to form heterodimers with TREs [26]. TRs undergo conformational changes both before and after T3 engagement, thus activating or repressing the transcriptional apparatus. When bound to ligands, TRs recruit coactivator complexes to target genes to activate transcription, whereas unbound TRs recruit corepressor complexes to target genes to repress their expression. Chromatin remodelling and histone modifications play a significant role in the regulation of genes by TRs [26]. All vertebrates have two TR genes, namely, TRα and TRβ [27]. Thyroid hormone has been the subject of most studies reporting on hormones involved in controlling ISCs. Mice, anurans and in vitro intestine organoid cultures are among the experimental models used to explore thyroid hormones.

#### Experiments on mice

Several mouse models with single TR or T3 or double TR/T3 knockouts have been developed to examine the critical role of TR in controlling T3 signalling during mammalian development and identify the intriguing properties and significant molecular pathways of adult ISCs. To date, adult mammalian ISCs have been extensively studied. Recent studies have reported that the development of adult ISCs in mice occurs during the neonatal period when plasma T3 levels are highest. T3 can increase the crypt size, enhance the proliferation of crypt cells and increase the pool of ISCs and Paneth cells in mouse models stimulated with T3 [28, 29]. Additionally, intestinal abnormalities and altered ISC proliferation are driven by mutations or deficits in mice T3 receptors. In addition to having shorter intestinal villi and more differentiated Paneth and cup cells in the crypt, with decreased crypt cell proliferation and fewer ISCs, adult mice with TR knockout can develop constipation owing to poor intestinal motility. Therefore, delayed replacement of intestinal epithelial cells and impaired proliferation/regeneration of ISCs may be caused by TR mutations [28].

#### Experiments on anurans

Owing to the dependence of a mammalian neonate on its mother and the uterine closure of the embryo, it is challenging to understand the significance of T3 in postembryonic development. Development of the anurans *Xenopus laevis* and *Xenopus tropicalis* is similar to the postembryonic development of mammals. Importantly, metamorphosis in anurans can be easily controlled by controlling the supply of T3 to tadpoles; therefore, anurans are very valuable models for studying organ maturation and ISC formation under the action of T3 during postembryonic development in vertebrates [30].

In the anuran *X. laevis* or *X. tropicalis*, the intestine undergoes significant modification during metamorphosis and resembles a basic tubular structure with a single epithelial fold in tadpoles. Thyroid hormone (TH) induces apoptosis in a majority of larval epithelial cells during differentiation. When TH is continuously expressed, the remaining epithelial cells (SC precursors) differentiate into ISCs, which de novo produce a multi-fold adult epithelium that resembles that of mammals and is encircled by thick layers of connective tissue and muscle [31]. Similar to mammals, anurans have two TR isoforms, namely, TRα and TRβ. TRα expression is only found in proliferating adult epithelial primordial cells produced by ISCs after the initiation of TH-dependent remodelling. As intestinal folds develop, TRα expression concentrates in the valleys of the folds, where ISCs are found [32, 33]. In contrast, as endogenous TH levels increase, TRβ expression is momentarily increased throughout the gut, with the adult epithelium primordium exhibiting the highest increase. TRβ is expressed on both ISCs and larval epithelial cells, whereas TRα is expressed on SC precursors but not on larval cells proper that are ready to undergo apoptosis [34]. Different organs express TRα and TRβ mRNAs differently. The expression of TRα mRNA is widespread during the larval and metamorphic stages and is primarily seen in certain adult organs, such as the hindlimbs [35]. However, TRβ mRNA is momentarily elevated during the peak of metamorphosis along with an increase in endogenous TH levels, and it is mostly expressed in organs unique to larvae, such as the tail and gills [36]. These results suggest that TRα and TRβ play different roles in intestinal larval-to-adult remodelling [36].

TRα controls the subsequent development and/or maintenance of ISCs and the dedifferentiation of SC precursors into ISCs. The lack of TRα delays these processes as observed in TRα-knockout tadpoles [37]. TRα deficiency prevents intestinal remodelling in *X. tropicalis* during T3-stimulated development, indicating that TRα is necessary to initiate gut remodelling. After T3 treatment, cell proliferation gradually increases in the gut of wild-type tadpoles but not in TRα-KO tadpoles [38]. Additionally, the gut of wild-type tadpoles has enhanced expression of the stem cell marker gene Lgr5, whereas this upregulation is repressed or delayed in TRα-KO tadpoles [39]. Therefore, TRα may play a role in regulating the proliferation of ISCs. Furthermore, high levels of apoptotic signals are observed in the intestinal epithelial cells of wild-type tadpoles but not in those of TRα-KO tadpoles after 2 days of T3 treatment, indicating that TRα is necessary for T3-induced apoptosis. A majority of larval epithelial cells undergo apoptosis with increased levels of T3 during intestinal metamorphosis [40]. Activation and promotion of cell cycle-related genes by TRα are crucial during intestinal metamorphosis. In addition, TRα may play an important role in remodelling the extracellular matrix by metalloproteinases (MMPs), which is induced by T3 in larval epithelial cells [39]. TRα mediates T3 during intestinal metamorphosis and ISC maintenance. However, TRα knockdown inhibits T3-induced intestinal remodelling and significantly reduces the apoptosis and proliferation of adult stem cells after prolonged T3 treatment.

TRβ may play a role in the formation of ISCs after their emergence and the death of larval cells. Although TRβ expression is briefly and significantly upregulated during gut metamorphosis, there are only very minor variations in gut remodelling between thyroid hormone receptor β knockout (TRβ-KO) and wild-type tadpoles [41]. Contrary to TRα, which is expressed at high levels from the pre-metamorphosis stage to the end of metamorphosis, TRβ is expressed at very low levels before metamorphosis but is triggered as a direct target gene of T3, peaking at the end of metamorphosis. According to external morphology, TRβ KO does not affect tadpole development before metamorphosis but delays tail regression during metamorphosis. In *X. tropicalis*, TRβ KO has relatively minimal effects on hindlimb development and gut remodelling. However, T3-induced intestinal remodelling, including reduced length, adult stem cell development and proliferation, and larval epithelial cell death were inhibited by TRβ knockout. These facts suggest that TRβ has an effect on the intestinal remodelling process [42].

#### Organoid cultures

Reconstituted organoid cultures and 3D primary intestinal epithelial organoid cultures are excellent in vitro models to study ISCs. Studies employing these models have validated the involvement of T3 and TR in the development of adult stem cells during intestinal metaphase [43]. T3 can control cell proliferation in organoid cultures. Organoids treated with T3 have longer and larger buds and faster cell cycles, resulting in the earlier emergence of buds during in vitro growth. The loss of ISCs and their differentiation to intestinal secretory cells can increase the turnover [44]. Intestinal Lgr5 + crypt cells exclusively express TRα, which directly activates the Wnt and Notch pathways and promotes cell growth in these cells. TRβ, which is encoded by the TRβ gene, is only expressed on differentiated epithelial cells of the villi; however, its function in the gut remains unknown. In vitro organoid dysplasia and reduced stem cell activity result from the loss of a specific TRα function, and TRβ-KO organoids do not exhibit any similar symptoms. In addition, it has been demonstrated that the absence of TRα but not TRβ reduces ISCs activity [45].

#### Mechanisms underlying thyroid hormone control of intestinal stem cells

Identifying pathways involved in TR control in the gut and the related target genes is essential for understanding the regulatory role of T3 in ISCs. During intestinal remodelling, T3 can stimulate numerous signalling pathways crucial for the proliferation and function of stem cells. These pathways include the Notch, WNT, hedgehog and BMP signalling pathways [46–50]. However, further research is required to determine the effects of these signalling pathways on the growth of adult ISCs.

**Fig. 2:**
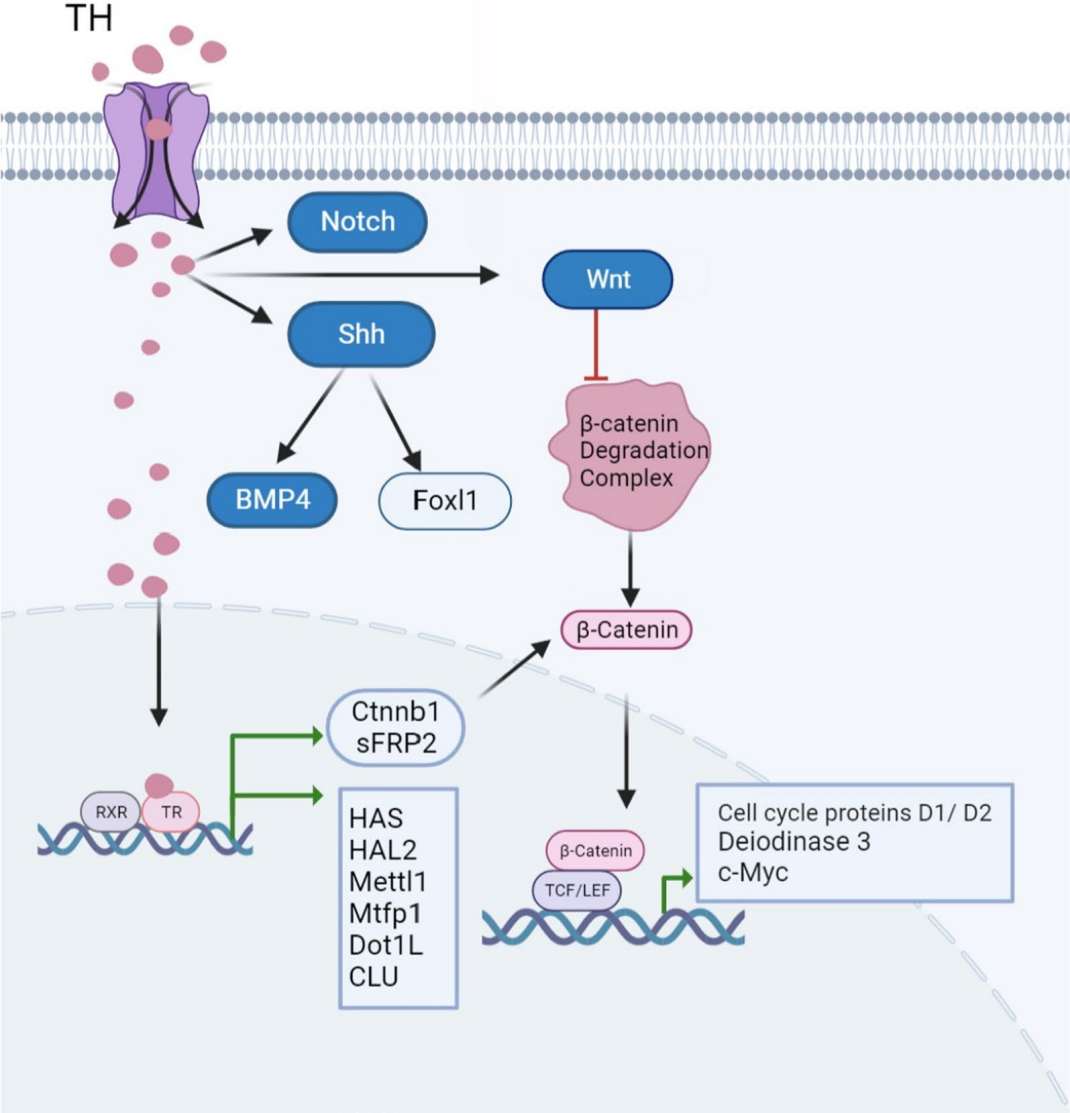
A molecular model of the action of thyroid hormone (TH) on the intestinal stem cell pathway was proposed. In intestinal stem cells, TH enters the nucleus and binds to TR to directly regulate the expression of HAS, HAL2, Mettl1, Mtfp1, DotiL and CLU genes. In addition, TH regulates the expression of multiple targets involved in the Wnt, Notch and hedgehog pathways. In the Wnt signalling pathway, the binding of TH to TR positively regulates the expression of Ctnnb1 and sFRP2 genes. In turn, Ctnnb1 and sFRP2 proteins allow β-catenin translocation to the nucleus by interacting with Wnt/β-catenin, where β-catenin forms a complex with transcription factors TCF/LEF, leading to increased expression of Wnt target genes, such as Cell cycle proteins D1/D2, Deiodinase 3 and c-Myc. In hedgehog signalling pathways, TH can act directly on Shh and promote enhanced transcription and expression of BMP4 and Foxl1

Numerous T3-regulated targets play a role in intestinal remodelling and the generation and proliferation of ISCs (Fig. 2). In the ISCs and surrounding connective tissue cells of adult *X. laevis*, TH can upregulate hyaluronate synthase (HAS), which is implicated in the development of ISCs through newly synthesised hyaluronic acid (HA). During the late stage of vertebrate embryonic development and maturation, signal transduction is important for controlling ISCs [51]. LGR5, a marker for ISCs, is inhibited via inhibition of HA production [52]. HAL2 is selectively produced during metaplasia of developing/proliferating adult ISCs in *X. laevis*, and T3 can induce HAL2 during the early stage of adult stem cell development. The histidine gene in HAL2 is the first enzyme in the histidine catabolic pathway, which breaks down histidine into ammonia and uridine. Therefore, histidine catabolism may play a role in the formation of adult stem cells [53]. The Ctnnb1 gene, which produces a β-catenin protein, is directly regulated by TRα through transcription. The cell cycle proteins D1 and D2 and c-Myc are some targets that are activated as a result of increased expression of β-catenin protein [54]. Additionally, secreted frizzled-related protein (sFRP2) is a direct target of TRs and positively controls the common WNT pathway in intestinal progenitor cells in vitro [55]. In *X. tropicalis*, the Myc/Mad/Max axis is involved in the development and/or proliferation of adult stem cells and the death of larval epithelial cells. Both Mad1 and c-Myc heterodimerise with MAX and bind to the same target genes with contrasting consequences. Mad suppresses the expression of c-Myc target genes and is associated with quiescence or cell differentiation, whereas c-Myc is a well-known oncogene that increases target gene transcription and promotes cell proliferation. During intestinal metamorphosis, Mad1 and c-Myc are expressed on various epithelial cells, with Mad1 expression being high on apoptotic larval epithelial cells and c-Myc expression being high on proliferative adult stem cells [31]. During metamorphosis, TRs can directly activate Mettl1 at the transcriptional level through the TRE of the promoter region in the gut. During intestinal remodelling, Mettl1 modulates target tRNAs to affect translation, thus promoting the generation and/or proliferation of stem cells [56]. The Mtfp1 gene is directly activated by T3 through the TRE of introns, and this activation influences mitochondrial fission, which in turn stimulates the growth and/or proliferation of adult ISCs [57]. Forkhead box l1 (Foxl1) expression is indirectly upregulated by TH via Shh signalling, and organ-autonomous induction of Foxl1-expressing cells by TH occurs simultaneously with the emergence of stem cells in the tadpole gut in vitro. Recent studies have reported that Foxl1-expressing mesenchymal cells are an important component of the intestinal niche. Intestinal niche cells that express Foxl1 are evolutionarily conserved in terrestrial vertebrates and can stimulate the formation of stem cells during amphibian metamorphosis by activating TH/Shh signalling [58]. PRMT1 is a well-known TR co-activator and a histone H4R3 methyltransferase. It is induced in response to T3 during intestinal metamorphosis in *X. laevis*. In developing stem cells, T3 appears to activate the transcription factor cMyc, and cMyc, in turn, appears to activate the PRMT1 promoter. Therefore, T3 can indirectly increase PRMT1 expression, and hat PRMT1 can act as a TR co-activator to further enhance T3 signalling and support the formation of ISCs [59]. Dot1L, a histone methyltransferase, is the only enzyme that methylates histone H3K79 in vitro and is directly regulated by T3 at the transcriptional level through the binding of TR to the TRE of its promoter. It enhances transcriptional activation of TR and functions as a TR coactivator through a positive feedback mechanism during intestinal remodelling and adult stem cell development [60]. In a study, RNA-sequencing analysis revealed that the clusterin (Clu) gene, which is associated with the suppression of the cycling/quiescent/revitalised population of ISCs, was an important upregulated differentially expressed gene (DEG) associated with SC characteristics in T3-treated organoids [45]. It is noteworthy that TH has a different mechanism of action on colon cancer stem cells than on small intestine stem cells. Thyroid hormones control the balance between proliferation and differentiation of colon cancer stem cells (CSC), specifically, TH activates the Wnt/β-catenin pathway, while deiodinase 3 (D3) is a direct target of β-catenin, which binds to the D3 promoter and activates D3 transcription, ultimately promoting the proliferation of colon cancer stem cells [61].

### Glucagon-like peptide-2

Glucagon-like peptide 2 (GLP-2) is produced by L cells in the gastrointestinal tract and maintains the dynamic homeostasis of intestinal epithelial cells. It is an intestinal hormone comprising 33 amino acids derived from proglucagon cleaved by protein hydrolase 1/3 [62]. It can exert direct and indirect effects on responding cells. Cells that respond to GLP-2 include enterocytes, cup cells, neurons, subepithelial myofibroblasts, endothelial cells and certain enteroendocrine cells [63]. The effects of GLP-2 on cells located near the intestine are mediated by signalling molecules released from cells with surface expression of the GLP-2 receptor (GLP-2R). When GLP-2 binds to its receptor (GLP-2R), it initiates signals that promote the proliferation of intestinal crypt cells, thereby promoting intestinal growth and improving intestinal digestion, absorption and barrier function. GLP-2R is a G protein-coupled receptor localised in cells within the lamina propria and scattered neurons of the enteric nervous system. Its activation on subepithelial myofibroblasts leads to the release of growth factors, mainly insulin-like growth factors 1 and 2, keratinocyte growth factor, epidermal growth factor and transforming growth factor-beta. Through this paracrine effect, GLP-2 can expand different cells in the intestine [64]. To overcome the short half-life of the natural GLP-2, a degradable GLP-2 analogue (h[Gly2]-GLP-2) named teduglutide has been developed. Teduglutide has been approved for the chronic treatment of short bowel syndrome in the United States of America and Europe based on a placebo-controlled phase 3 study and is used to reduce the requirement of parenteral nutrition while increasing the size and absorption of the intestinal epithelium [65].

In mice with acute graft-versus-host disease (GVHD), Norona et al. used the GLP-2 analogue teduglutide [66] and discovered that GLP-2 treatment overcame the deficiency in ISCs caused by graft-versus-host disease and encouraged ISC regeneration. In addition, injection of 500-nM teduglutide significantly increased the surface area of intestinal organoids [66]. In another study, immunofluorescent-labelled mice with GVHD were examined using confocal microscopy to evaluate whether GLP-2 treatment offers protection against GVHD by increasing the number of ISCs. The results demonstrated that teduglutide treatment increased the number of Lgr5 + ISCs, which counteracted the effects of GVHD on the number of Lgr5 + ISCs. Furthermore, qPCR was used to examine the expression of the stem cell marker Olfm4. Compared with mice treated with a vehicle, those treated with teduglutide had high Olfm4 expression. Additionally, mice treated with teduglutide and developing GVHD had increased expression of the stem cell marker prominin-1 (Prom1). These results indicate that GLP-2 possesses anti-apoptotic, histo-protective and regenerative properties, and the GLP-2 analogue teduglutide increases the number of ISCs and the expression of ISC markers during GVHD. In addition, reduced expression of the apoptosis execution factor caspase 3, downregulated transcription of several genes associated with cell death and increased transcription of the KGF gene, a gene associated with growth factors, are some processes that were demonstrated in the study [66].

In a study by Chen et al. [67], acute GLP-2 administration significantly enhanced the progression of ISCs from the G1 phase to the S phase, and long-term GLP-2 treatment increased ISC expansion, which may be dependent on IGF-1. Lgr5-enhanced green-fluorescent protein-internal ribosome entry site-Cre recombinase-oestrogen receptor T2 (eGFP-IRES-creERT2) mice were administered teduglutide or a vehicle 6 and 3 h before the mice were sacrificed. S-phase cells were labelled with EDU 1 h before the mice were sacrificed. Acute treatment with teduglutide increased the proportion of S-phase ISCs, whereas treatment with a GLP-2 antagonist decreased the proportion of S-phase ISCs. After GLP-2 treatment, transcriptional analysis of the entire jejunal and colonic sections using gene microarrays revealed that MCM3 expression was elevated in both the jejunum and colon. This information was used to identify potential targets that may mediate the proliferative effects of GLP-2 on the intestine. The stimulatory effects of GLP-2 on the number of ISCs were diminished in intestinal epithelial (IE)-IGF-1R-KO mice. Therefore, GLP-2 substantially stimulates the advancement of S-phase Lgr5 + /Olfm4 + ISCs via GLP-2R, and MCM3 may be a potential target for its proliferative effects. In addition, GLP-2 persistently increases the number of ISCs in an IGF-1R-dependent manner [67]. In a study by Bradley et al. [68], GLP-2 increased the length of the functional crypt-villus axis by promoting the proliferation of crypt cells and inhibiting the apoptosis of epithelial cells. The pro-intestinal effects of GLP-2 may depend on the expression of the BMI-1 gene, and the role of BMI-1 in the proliferation of TA cells and/or preventing their differentiation may be affected by GLP-2.

In conclusion, GLP-2 promotes ISC regeneration, inhibits apoptosis, exerts histo-protective and regenerative effects on the intestine, dilates intestinal organs, lengthens the functional crypt-villus axis and stimulates ISC proliferation. Teduglutide, an anti-degradative GLP-2 analogue, is used in clinical practice and improves enteral nutrition and intestinal growth in patients with short bowel syndrome [69]. Related mechanisms include downregulation of the transcription of several cell death-related genes by GLP-2, including decreased expression of caspase 3; promotion of proliferation as evidenced by the promotion of upregulation of the growth factor-related gene KGF and BMI-1; and induction of the progression of S-phase ISCs through the action of GLP-2R on the MCM3 gene, which in turn stimulates ISC expansion. To date, a few studies have examined the role of GLP-2 in Lgr5 + ISCs. Therefore, further investigation should be performed using organoid models that resemble intestinal exosomes.

### Androgens

Androgens (from the Greek root word ‘andro,’ meaning male or man) are typical male hormones that maintain masculine characteristics by activating the androgen receptor (AR), a ligand-induced nuclear receptor that, when activated, serves as a transcription factor [70]. The primary androgen in men is testosterone (T, androst-4-ene-17-ol-3-one), which is produced by mesenchymal cells in the testis and delivered into the bloodstream. When T is converted to the most potent natural androgen, 5-dihydrotestosterone (DHT, 5-androst-17-ol-3-one), the androgen signal is further enhanced in a few selected target tissues [71].

DHT activates AR in primary stromal cells in vitro, resulting in the promotion of organoid development. In a study on male mice, the number of enterocytes and intestinal secretory epithelial-like cells was increased and that of S-phase cells was decreased after 2 weeks of treatment with AR antagonists, which prevented the proliferation of ISCs. However, AR agonists prevented ISC differentiation and enhanced ISC proliferation in mice [71]. Therefore, androgens can simultaneously decrease the number of enterocytes and intestinal secretory lineage cells and increase the proliferation of ISCs, thereby promoting the growth of intestinal organs.

Mechanistically, the BMP pathway prevents and the Wnt pathway promotes ISC proliferation. Androgens can promote the growth of ISCs by activating the Wnt pathway and suppressing the BMP pathway. In particular, they can downregulate the Wnt-related antagonists Dkk2, Dkk3 and Sfrp1 and the BMP signalling-related proteins Bmp4 and Tgfb1 and upregulate b-catenin, a key component of the Wnt pathway, and the BMP signalling antagonists angptl2 and chrd. Additionally, AR expression is higher on intestinal mesenchymal cells than in the crypt foci, and androgens can influence stromal cells to promote crypt cell proliferation. Therefore, androgens simultaneously promote the proliferation of ISCs and suppress the development of intestinal epithelial cells by upregulating Wnt signalling and negatively regulating BMP signalling in stromal cells [72]. The increased incidence of colon cancer among men may be explained by these findings.

### Insulin

Insulin is a crucial hormone that controls glucose levels and is involved in the control of energy metabolism throughout the body [73]. In addition, it controls several signalling pathways. The IIS pathway controls various processes in multicellular organisms, including ageing, reproduction, nutrition, metabolism, stress resistance and growth [74]. High insulin levels in obesity-induced stem cell niches influence ISCs, thereby regulating organ size. Insulin can promote the proliferation of crypt cells, including ISCs, and several detrimental effects on tissue function are associated with these proliferative effects. Increased proliferation increases the likelihood of developing mutations and restricts the development of DNA repair mechanisms, which may promote the formation of tumours and cancer and contribute to the increased risk of bowel cancer associated with obesity [75].

The IIS pathway plays a survival-sustaining role in the ISCs of *Drosophila melanogaster*. The midgut of adult Drosophila is an appealing model for research into the regulation of stem cell maintenance and proliferation because it has several features similar to those of the mammalian gut. ISCs are found in the epithelial basement membrane in the midgut of Drosophila. They undergo symmetric division, with one daughter cell retaining its stemness and the other developing into an enteroblast (EB). The preservation of tissue homeostasis is significantly impacted by the control of self-renewal, proliferation and differentiation of ISCs. In a study, suppression of the IIS pathway via RNAi knockdown of the insulin receptor (InR) in ISCs reduced the lifespan of experimental flies compared with control flies and reduced their survival under starvation or malnutrition conditions. Additionally, the reproductive and eating capacities of these flies were diminished, and their bodies contained less glucose and glycogen. Therefore, InR knockdown can reduce the functional integrity of ISCs, which reduces the lifespan of flies and suggests that insulin is crucial for the normal function of stem cells via IIS [76].

The pancreas releases circulating insulin, which affects ISCs through mesenchymal cells below the crypt. ISCs express InR subtypes A and B. The activity of insulin-IGF-1 can be influenced by InR activity, and the IIS pathway positively controls the growth of ISCs. Mechanistically, the IIS pathway, when activated, regulates ISC proliferation by activating JAK–STAT and EGFR signalling in the gut [76]. However, the proliferation of ISCs induced by insulin is reduced when the PI3K/Akt pathway is inhibited [75].

### Leptin

Leptin was discovered as the first signalling protein produced by adipocytes (adipokines). It was first discovered in mouse adipocytes and is a peptide hormone produced by the OB gene. Leptin plays a critical role in maintaining body weight, hunger and energy balance and is primarily secreted into the bloodstream by adipocytes [77]. Recent studies have reported the association between leptin and control of the ISC milieu.

For the development and maintenance of intestinal homeostasis, pericrypt mesenchymal cells serve as the milieu for ISCs. The proliferation of ISCs near the base of the epithelial crypt is necessary for the regeneration of epithelial cells, and mesenchymal cells that surround these stem cells strictly control their maintenance and development. WNTs, gremlin (GREM) and BMP, which are crucial for intestinal renewal and stem cell maintenance, are secreted by several subpopulations of mesenchymal cells, such as intraepithelial myofibroblasts, GLI family zinc finger 1 + (Gli1+) fibroblasts and telocytes [78]. Leptin receptors are highly expressed on the surface of crypt MSCs, which suggests that endogenous leptin is an important factor in the control of MSC activity, especially in the crypt, based on the gene expression profile of crypt MSCs. A study showed that leptin stimulation increased the expression of the crypt ecological niche-associated factor Wnt2b in Lepr-deficient mice, indicating that leptin and WNT can stimulate stem cell proliferation. In addition, leptin was found to be involved in the proliferation of ISCs and the protection and repair of mucosal damage because the expression of Wnt2b was significantly lower in crypt mesenchymal cells than in controls. These results demonstrate the significance of Lepr signalling for the expression of Wnt2b, which is required for the survival and proliferation of ISCs [78].

In the colon, leptin can influence the biology of colorectal tumour stem cells. It was found that colorectal cancer stem cells express leptin receptor ObR and respond to leptin. Leptin activates extracellular signal-associated kinase (ERK) 1/2 and AKT signalling pathways and enhances cell proliferation. Blockade of ERK1/2 completely counteracts leptin-enhanced cell growth, suggesting that the ERK1/2 pathway is responsible for the increased proliferation of leptin-induced colorectal cancer stem cells [79].

### Growth hormone

Growth hormone (GH) is a pleiotropic hormone that is crucial for regulating several physiological processes. It promotes the expression of genes and intracellular signalling pathways by binding to the growth hormone receptor (GHR) [80]. GH can promote the proliferation of ISCs, formation of crypt-like organs, expression of ISC stemness markers in intestinal-like organs and differentiation of ISCs into Paneth cells and enterocytes. It primarily exerts anabolic effects by promoting the synthesis of IGF-1 [81].

In animal models, GH has been reported to influence intestinal tissue adaptability. Compared with control mice, transgenic mice overexpressing GH had increased body and intestinal weight [82]. Similarly, GH can affect the activity of ISCs or crypt cells. Intestinal shrinkage caused by GH deficiency after pituitary resection in rats is mostly attributed to decreased mitosis of epithelial ISCs. The size of intestinal epithelial cells, crypt volume and villi volume are lower in GH-deficient rats than in controls; and intraperitoneal administration of GH can restore these parameters to their normal levels [83]. Additionally, subcutaneous injection of bovine GH can increase the small intestine crypt depth/villi height ratio, thereby decreasing villi size and increasing crypt depth. GH can promote crypt cell proliferation, inhibit crypt cell differentiation and/or decrease the lifespan of epithelial cells, resulting in a higher crypt depth and lower villi size [84]. In a study, Cultivation of mouse crypt organs using the Matrigel culture method developed more intestinal crypt fossa-like organs after receiving GH treatment. Additionally, compared with control treatment, GH treatment increased the expression of stemness markers such as Lgr5, Bmi1, Msi1 and EphB3 in cultured mouse crypt organs. In cultured mouse crypt organs, GH altered the expression of differentiation markers, reduced the production of chromogranin A (a marker of enteroendocrine cells) and increased the expression of lysozymes and Ki67, indicating increased cell proliferation [85]. Another study reported that subcutaneous GH administration promoted the development of organs similar to crypt fossae and increased the expression of stemness markers such as Lgr5, Msi1 and EphB3 in mice [85]. These in vitro and in vivo studies indicate that GH promotes the differentiation of ISCs into Paneth cells and enterocytes, activates ISC proliferation, improves crypt organogenesis and increases the expression of ISC stemness markers in intestinal organs.

### Somatostatin

Somatostatin (SST) is a peptide hormone that is primarily produced by the central nervous system and endocrine cells. Specific varieties of neuroendocrine cells (NECs) express SST and SST type 1 receptor (SSTR1) [86]. These NECs are found next to colonic stem cells in the niches of crypt stem cells. The SST signalling pathway maintains colon CSC in a dormant state and prevents their multiplication through SSTR1 + NECs. Excess of intestinal stem cells in intestinal tumours may be attributed to aberrant SST signalling. In a study, acetaldehyde dehydrogenase (ALDH) was used to identify malignant colon CSC in colorectal cancer cell lines, and the proportion of SSTR1 + cells was negatively correlated with the proportion, proliferation and sphericity of malignant cells in the intestine. Furthermore, ALDH + colon cancer cells did not express SST, and sphere formation and proliferation abilities of ALDH + cells were suppressed after they were co-cultured with SSTR1 + cells. These findings indicate a feedback mechanism between colon CSC and NECs that helps to control colon CSC, and this feedback loop is regulated by SST signalling. Additionally, SST signalling manages NEC maturation, which aids in stem cell arrest and inhibition of cell proliferation [87].

### Corticotropin-releasing hormone

The hypothalamus releases corticotropin-releasing hormone (CRH), which promotes the pituitary gland to release the adrenocorticotropic hormone, which subsequently causes the adrenal glands to generate cortisol [88]. Additionally, CRH influences the function of intestinal cells, including immune cells, epithelial cells, enteric neurons and smooth muscle cells [89]. To date, two G protein-coupled CRH receptors, namely, CRHR1 and CRHR2, have been identified [90]. It is noteworthy that CRH plays dual roles in controlling mucosal damage. CRHR1 mediates intestinal injury by stimulating intestinal inflammation, increasing intestinal permeability, altering the intestinal shape and controlling intestinal flora in case of elevated stress. CRHR2 is crucial for intestinal regeneration because it activates ISCs, enhances colonic shape, lengthens crypts and increases the number of cup cells per crypt. In a study, selective blockade of CRHR1 and promotion of CRHR2 activity prevented the development of intestinal injury and enhanced repair in a mouse model of neonatal maternal isolation (MS) with an increased risk of intestinal injury such as necrotizing small bowel colitis in the neonatal period. Intestinal inflammation is mediated by NF-κB, a downstream mediator of CRHR1. Increased phosphorylation of STAT3 and IL-22, which is mediated by CRHR2, increases the number of Lgr5 + ISCs [91].

### Melatonin

N-acetyl-5-methoxytryptamine, often known as melatonin, is an endogenous hormone produced by the pineal gland and many tissues including the liver, gut and bone marrow. Melatonin primarily regulates sleep, neuroendocrine activity and circadian rhythm [92]. Recent studies have demonstrated that melatonin induces apoptosis and autophagy in colon cancer CSC by regulating the Oct4–PrPC axis, which has tumour-suppressing properties [93]. The expression of PrPC and Oct4 is strongly correlated with tumour stage and metastasis in colorectal cancer. Downregulation of PrPC caused by melatonin can prevent the expression of the stem cell markers Oct4, Nanog, Sox2 and ALDH1A1, which inhibits tumour development, proliferation and tumour-mediated angiogenesis by affecting ISCs [93].

#### Progastrin

Gastric G cells synthesise progastrin, which is produced by cleaving the C-terminus of the signalling peptide [94]. Progastrin is further processed to create gastrin, which is amidated and glycine-extended. Although progastrin and other non-amidated gastrin proteins normally constitute < 10% of the total released peptide, higher levels have been reported in some patients with gastrointestinal cancer [95]. In the mouse colonic mucosa, overexpression of human progastrin promotes cell proliferation and the development of colorectal cancer. Progastrin can bind to colon stem cells that express GPR56, which can promote colon growth by enhancing crypt fission and colon stem cell multiplication. The development of ISCs in response to progastrin is a key initiator of colorectal carcinogenesis [96, 97].

## Conclusion

**Fig. 3:**
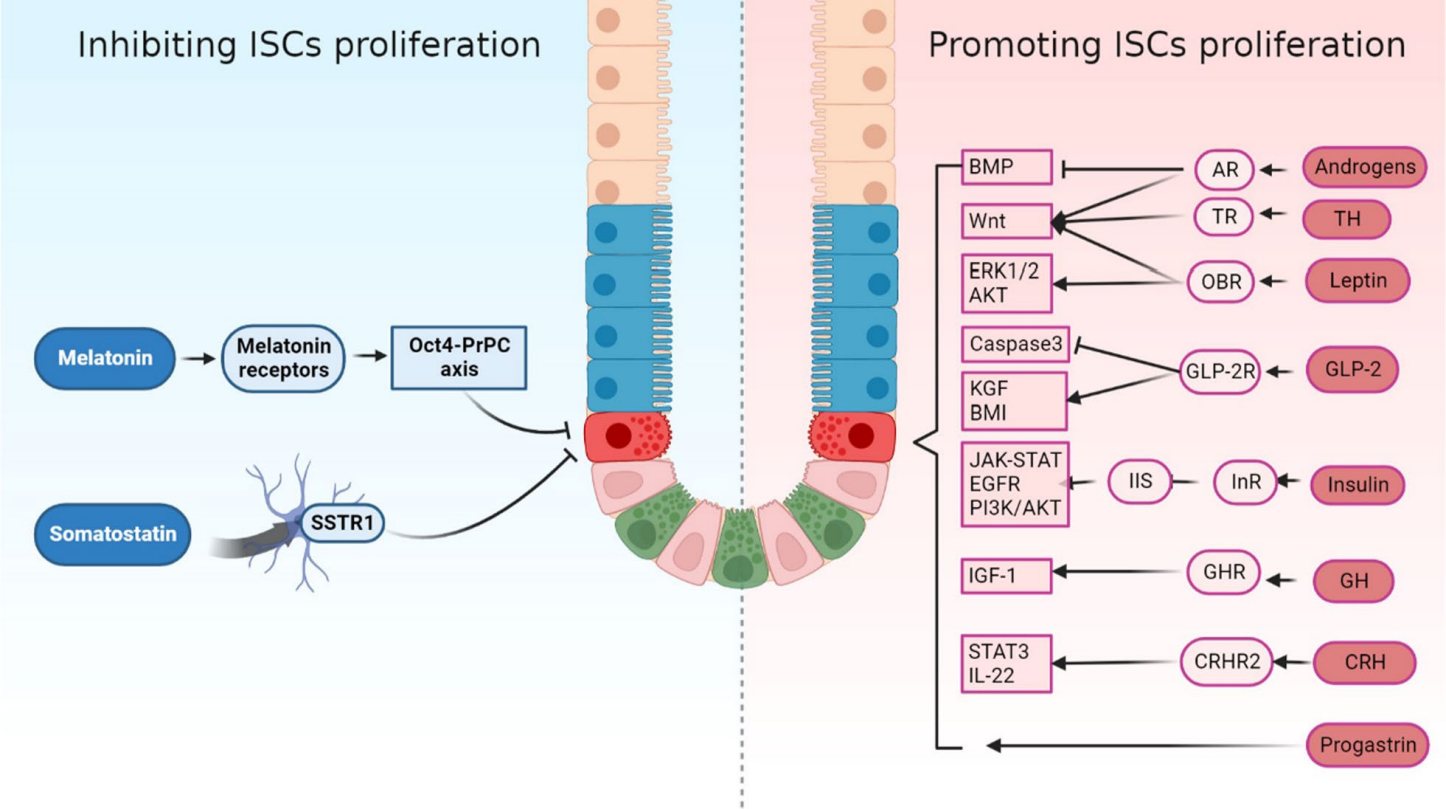
Functions of different hormones on intestinal stem cells and related mechanisms. On the left side of the diagram are two hormones capable of inhibiting the proliferation of intestinal stem cells. On the right side of the diagram are eight hormones that promote the proliferation of intestinal stem cells

The crypt-villus axis of the gut is intricate and important for the regeneration of the intestinal epithelium. ISCs located at the base of the crypt maintain intestinal homeostasis by multiplying and differentiating into various intestinal epithelial cells. The intestine is a vital endocrine organ and a site for numerous hormones to function in the body. This review summarised the effects of different hormones on the ability of stem cells to undergo proliferation and differentiation (Fig. 3). Among the hormones associated with intestinal stem cells, the most studied hormone is thyroid hormone. Thyroid hormone induces the proliferation of mature intestinal stem cells, initiates intestinal remodelling and controls the dedifferentiation of SC precursors into SCs and the development of SCs during metaplasia. Other hormones that promote the proliferation of ISCs include glucagon-like peptide-2, androgens, insulin, leptin and adrenocorticotropic hormone. Growth inhibitors and melatonin are examples of hormones that prevent the proliferation of ISCs. Hormones influence the renewal and proliferation of ISCs through several processes (Table 2). Notably, intestinal stem cells can be divided into small intestine and large intestine epithelial stem cells due to differences in morphology and function. The current study found that thyroid hormone, leptin, Somatostatin, melatonin and progastrin have specific mechanisms for the regulation of colonic epithelial stem cells. These differences may be used to explain why the prevalence of colorectal cancer is much higher than that of small bowel cancer. Theoretically, hormones can serve as targets for the treatment of intestinal illnesses because of their effects on ISCs.

**Table 2:** Effects and mechanisms of hormones in intestinal stem cells

Hormones	Action on intestinal stem cells	Mechanisms	References
Thyroid hormones	Stimulate the proliferation of intestinal stem cells in the mature intestine, initiate intestinal remodelling during metamorphosis and control the dedifferentiation of SC precursors into SCs and the development of SCs	Upregulation of the HA/CD44 signalling pathway	[51, 52]
		Upregulation of the HAL2 gene	[53]
		Upregulation of the Ctnnb1 gene, which encodes a beta-linked protein and activates targets such as cyclins D1 and D2 and c-Myc	[55]
		Upregulation of the sFRP2 gene activates the WNT pathway	[55]
		Activation of the Myc/Mad/Max axis	[31]
		Activation of the Mettl1 gene	[56]
		Upregulation of the Mtfp1 gene	[57]
		Foxl1 expression is indirectly upregulated by thyroxine through Shh signalling	[58]
		Activation of the transcription factor cMyc in stem cells; cMyc in turn activates the PRMT1 gene	[59]
		Dot1L acts as a TR coactivator via a positive feedback mechanism	[60]
		Upregulation of the CLU gene	[45]
		Activation of D3 transcription via the Wnt/β-catenin pathway	[61]
Glucagon-like peptide 2	Stimulates ISC proliferation, dilates intestinal organs and resists intestinal cell apoptosis	Promotes upregulation of the growth factor-related gene KGF and decreases the expression of the apoptosis execution factor caspase 3	[66]
		Stimulates ISC expansion by inducing S-phase cell cycle progression in ISCs via GLP-2R; the cell cycle control gene MCM3 is a target for proliferative effects	[67]
		The role of GLP-2 in promoting intestinal stem cells requires the expression of the BMI-1 gene	[68]
Androgens	Promotes the proliferation of intestinal stem cells and inhibits the differentiation of intestinal epithelial cells	Upregulation of the Wnt pathway and downregulation of the BMP pathway	[72]
Insulin	Promotes the proliferation of intestinal stem cells	Regulation of ISC proliferation through insulin–IGF-1 signalling (IIS) activation of JAK–STAT, PI3K/Akt and EGFR signalling in the intestine	[75, 76]
Leptin	Involved in the proliferation of intestinal stem cells	Upregulation of the crypt ecological niche-associated factor Wnt2b	[78]
		Activation of ERK 1/2 and AKT signalling pathways to enhance stem cell proliferation	[79]
Growth hormones	Activates the proliferation of intestinal stem cells and drives the differentiation of ISCs to Paneth cells and enterocytes	Stimulates the production of IGF-1	[81–85]
Somatostatin	Contributes to stem cell arrest and inhibition of proliferation	SST signalling controls SSTR1 + neuroendocrine cells, which regulate intestinal stem cells through a paracrine mechanism	[87]
Corticotropin-releasing hormone	Activates intestinal stem cells, increases crypt length, improves colonic morphology and promotes intestinal repair	Increased phosphorylation of STAT3 and IL-22 is mediated by CRHR2, leading to an increase in the number of intestinal stem cells	[91]
Melatonin	Induces apoptosis and autophagy in colon cancer intestinal stem cells	Adjusting the Oct4–PrPC axis	[93]
Progastrin	Promotes colonic stem cell expansion and crypt fission	Binding to GPR56-expressing colon stem cells	[96, 97]

## Data Availability

Please contact the corresponding author for data requests.

## Abbreviations

ISCs: Intestinal stem cells
TA: Transit-amplifying
SI: Small intestine
LI: Large intestine
LGR5: Leucine-rich repeat-containing G-protein-coupled receptor 5
Olfm4: Olfactomedin-4
CBC: Circulating crypt base columnar
Msi1: Musashi-1
Bmi1: B cell-specific Moloney murine leukaemia virus integration site 1
EphB3: Ephrin receptor-B3
c-Myc: C-Myelocytomatosis
Lrig1: Leucine-rich repeats and immunoglobulin-like domain protein 1
BMP2: Bone morphogenetic protein 2
BMP4: Bone morphogenetic protein 4
IIS: Insulin–IGF-1 signalling
T3: Thyroid hormones
TRH: Thyrotropin-releasing hormone
TSH: Thyroid-stimulating hormone
TRs: Hormone receptors
TREs: T3 response elements
RXRs: 9-Cis-retinoic acid receptors
TH: Thyroid hormone
TRβ-KO: Thyroid hormone receptor β knockout
HAS: Hyaluronate synthase
HA: Hyaluronic acid
sFRP2: Secreted frizzled-related protein
Foxl1: Forkhead box l1
Clu: Clusterin
CSC: Cancer stem cells
D3: Deiodinase 3
GLP-2: Glucagon-like peptide 2
GVHD: Graft-versus-host disease
Prom1: Prominin-1
AR: Androgen receptor
EB: Enteroblast
InR: Insulin receptor
GH: Growth hormone
SST: Somatostatin
SSTR1: SST type 1 receptor
NECs: Neuroendocrine cells
CRH: Corticotropin-releasing hormone

## References

[1] Julio-Pieper M, Bravo JA (2016). Intestinal barrier and behavior. Int Rev Neurobiol.

[2] Baulies A, Angelis N, Li VSW (2020). Hallmarks of intestinal stem cells. Development.

[3] Johnson LR (1977). Gastrointestinal hormones and their functions. Annu Rev Physiol.

[4] Thomas RP, Hellmich MR, Townsend CM (2003). Role of gastrointestinal hormones in the proliferation of normal and neoplastic tissues. Endocr Rev.

[5] Azkanaz M, Corominas-Murtra B, Ellenbroek SIJ (2022). Retrograde movements determine effective stem cell numbers in the intestine. Nature.

[6] Beumer J, Clevers H (2021). Cell fate specification and differentiation in the adult mammalian intestine. Nat Rev Mol Cell Biol.

[7] Sato T, Van Es JH, Snippert HJ (2011). Paneth cells constitute the niche for Lgr5 stem cells in intestinal crypts. Nature.

[8] Gehart H, Clevers H (2019). Tales from the crypt: new insights into intestinal stem cells. Nat Rev Gastroenterol Hepatol.

[9] Ludikhuize MC, Meerlo M, Gallego MP (2020). Mitochondria define intestinal stem cell differentiation downstream of a FOXO/Notch axis. Cell Metab.

[10] Bankaitis ED, Ha A, Kuo CJ (2018). Reserve stem cells in intestinal homeostasis and injury. Gastroenterology.

[11] Takahashi T, Fujishima K, Kengaku M (2021). Modeling intestinal stem cell function with organoids. Int J Mol Sci.

[12] Muñoz J, Stange DE, Schepers AG (2012). The Lgr5 intestinal stem cell signature: robust expression of proposed quiescent '+4' cell markers. Embo J.

[13] Metcalfe C, Kljavin NM, Ybarra R (2014). Lgr5+ stem cells are indispensable for radiation-induced intestinal regeneration. Cell Stem Cell.

[14] Yan KS, Chia LA, Li X (2012). The intestinal stem cell markers Bmi1 and Lgr5 identify two functionally distinct populations. Proc Natl Acad Sci U S A.

[15] Holmberg J, Genander M, Halford MM (2006). EphB receptors coordinate migration and proliferation in the intestinal stem cell niche. Cell.

[16] Liang X, Duronio GN, Yang Y (2022). An enhancer-driven stem cell-like program mediated by SOX9 blocks intestinal differentiation in colorectal cancer. Gastroenterology.

[17] Ji Y, Kumar R, Gokhale A (2022). LRIG1, a regulator of stem cell quiescence and a pleiotropic feedback tumor suppressor. Semin Cancer Biol.

[18] Kurokawa K, Hayakawa Y, Koike K (2020). Plasticity of intestinal epithelium: stem cell niches and regulatory signals. Int J Mol Sci.

[19] Qi Z, Chen Y (2014). Fate regulation of small intestinal stem cells. China Sci Life Sci.

[20] Lindemans CA, Calafiore M, Mertelsmann AM (2015). Interleukin-22 promotes intestinal-stem-cell-mediated epithelial regeneration. Nature.

[21] Zhou J, Valentini E, Boutros M (2021). Microenvironmental innate immune signaling and cell mechanical responses promote tumor growth. Dev Cell.

[22] Herrera SC, Bach EA (2019). JAK/STAT signaling in stem cells and regeneration: from Drosophila to vertebrates. Development.

[23] Jiang H, Tian A, Jiang J (2016). Intestinal stem cell response to injury: lessons from Drosophila. Cell Mol Life Sci.

[24] Carvalho DP, Dupuy C (2017). Thyroid hormone biosynthesis and release. Mol Cell Endocrinol.

[25] Mendoza A, Hollenberg AN (2017). New insights into thyroid hormone action. Pharmacol Ther.

[26] Allison LA (2021). Getting there: thyroid hormone receptor intracellular trafficking. J Biol Chem.

[27] Shi YB (2021). Life without thyroid hormone receptor. Endocrinology.

[28] Bao L, Roediger J, Park S (2019). Thyroid hormone receptor alpha mutations lead to epithelial defects in the adult intestine in a mouse model of resistance to thyroid hormone. Thyroid.

[29] Xi Y, Zhang D, Liang Y (2022). Proteomic analysis of the intestinal resistance to thyroid hormone mouse model with thyroid hormone receptor alpha mutations. Front Endocrinol (Lausanne).

[30] Ruthsatz K, Dausmann KH, Drees C (2020). Altered thyroid hormone levels affect the capacity for temperature-induced developmental plasticity in larvae of *Rana temporaria* and *Xenopus laevis*. J Therm Biol.

[31] Shi YB, Shibata Y, Tanizaki Y (2021). The development of adult intestinal stem cells: insights from studies on thyroid hormone-dependent anuran metamorphosis. Vitam Horm.

[32] Buchholz DR, Shi YB (2018). Dual function model revised by thyroid hormone receptor alpha knockout frogs. Gen Comp Endocrinol.

[33] Wen L, Shi YB (2016). Regulation of growth rate and developmental timing by Xenopus thyroid hormone receptor α. Dev Growth Differ.

[34] Choi J, Moskalik CL, Ng A (2015). Regulation of thyroid hormone-induced development in vivo by thyroid hormone transporters and cytosolic binding proteins. Gen Comp Endocrinol.

[35] Nakajima K, Tazawa I, Yaoita Y (2018). Thyroid hormone receptor α- and β-knockout *Xenopus tropicalis* tadpoles reveal subtype-specific roles during development. Endocrinology.

[36] Hasebe T, Fujimoto K, Buchholz DR (2020). Stem cell development involves divergent thyroid hormone receptor subtype expression and epigenetic modifications in the Xenopus metamorphosing intestine. Gen Comp Endocrinol.

[37] Choi J, Ishizuya-Oka A, Buchholz DR (2017). Growth, development, and intestinal remodeling occurs in the absence of thyroid hormone receptor α in tadpoles of *Xenopus tropicalis*. Endocrinology.

[38] Tanizaki Y, Zhang H, Shibata Y (2022). Thyroid hormone receptor α controls larval intestinal epithelial cell death by regulating the CDK1 pathway. Commun Biol.

[39] Tanizaki Y, Shibata Y, Zhang H (2021). Analysis of thyroid hormone receptor α-knockout tadpoles reveals that the activation of cell cycle program is involved in thyroid hormone-induced larval epithelial cell death and adult intestinal stem cell development during *Xenopus tropicalis* metamorphosis. Thyroid.

[40] Okada M, Wen L, Miller TC (2015). Molecular and cytological analyses reveal distinct transformations of intestinal epithelial cells during Xenopus metamorphosis. Cell Biosci.

[41] Nakajima K, Tanizaki Y, Luu N (2020). Comprehensive RNA-Seq analysis of notochord-enriched genes induced during *Xenopus tropicalis* tail resorption. Gen Comp Endocrinol.

[42] Shibata Y, Tanizaki Y, Shi YB (2020). Thyroid hormone receptor beta is critical for intestinal remodeling during *Xenopus tropicalis* metamorphosis. Cell Biosci.

[43] Ishizuya-Oka A (2017). Organ culture of the Xenopus tadpole intestine. Cold Spring Harb Protoc.

[44] Maher SK, Wojnarowicz P, Ichu TA (2016). Rethinking the biological relationships of the thyroid hormones, l-thyroxine and 3,5,3'-triiodothyronine. Comp Biochem Physiol Part D Genomics Proteomics.

[45] Godart M, Frau C, Farhat D (2021). Murine intestinal stem cells are highly sensitive to modulation of the T3/TRα1-dependent pathway. Development.

[46] Ishizuya-Oka A, Hasebe T (2008). Sonic hedgehog and bone morphogenetic protein-4 signaling pathway involved in epithelial cell renewal along the radial axis of the intestine. Digestion.

[47] Clevers H, Loh KM, Nusse R (2014). Stem cell signalling. An integral program for tissue renewal and regeneration: Wnt signaling and stem cell control. Science.

[48] Frau C, Godart M, Plateroti M (2017). Thyroid hormone regulation of intestinal epithelial stem cell biology. Mol Cell Endocrinol.

[49] Hasebe T, Fujimoto K, Kajita M (2017). Thyroid hormone-induced activation of notch signaling is required for adult intestinal stem cell development during *Xenopus laevis* metamorphosis. Stem Cells.

[50] Ishizuya-Oka A, Ueda S, Amano T (2001). Thyroid-hormone-dependent and fibroblast-specific expression of BMP-4 correlates with adult epithelial development during amphibian intestinal remodeling. Cell Tissue Res.

[51] Hasebe T, Fujimoto K, Kajita M (2017). Essential roles of thyroid hormone-regulated hyaluronan/CD44 signaling in adult stem cell development during *Xenopus laevis* intestinal remodeling. Stem Cells.

[52] Fujimoto K, Hasebe T, Kajita M (2018). Expression of hyaluronan synthases upregulated by thyroid hormone is involved in intestinal stem cell development during *Xenopus laevis* metamorphosis. Dev Genes Evol.

[53] Luu N, Fu L, Fujimoto K (2017). Direct regulation of histidine Ammonia-Lyase 2 gene by thyroid hormone in the developing adult intestinal stem cells. Endocrinology.

[54] Tanabe S, Aoyagi K, Yokozaki H (2016). Regulation of CTNNB1 signaling in gastric cancer and stem cells. World J Gastrointest Oncol.

[55] Sirakov M, Kress E, Nadjar J (2014). Thyroid hormones and their nuclear receptors: New players in intestinal epithelium stem cell biology?. Cell Mol Life Sci.

[56] Na W, Fu L, Luu N (2020). Direct activation of tRNA methyltransferase-like 1 (Mettl1) gene by thyroid hormone receptor implicates a role in adult intestinal stem cell development and proliferation during *Xenopus tropicalis* metamorphosis. Cell Biosci.

[57] Na W, Fu L, Luu N (2020). Thyroid hormone directly activates mitochondrial fission process 1 (Mtfp1) gene transcription during adult intestinal stem cell development and proliferation in *Xenopus tropicalis*. Gen Comp Endocrinol.

[58] Hasebe T, Fujimoto K, Ishizuya-Oka A (2020). Thyroid hormone-induced expression of Foxl1 in subepithelial fibroblasts correlates with adult stem cell development during Xenopus intestinal remodeling. Sci Rep.

[59] Xue L, Bao L, Roediger J (2021). Protein arginine methyltransferase 1 regulates cell proliferation and differentiation in adult mouse adult intestine. Cell Biosci.

[60] Fu L, Yin J, Shi YB (2019). Involvement of epigenetic modifications in thyroid hormone-dependent formation of adult intestinal stem cells during amphibian metamorphosis. Gen Comp Endocrinol.

[61] Cicatiello AG, Ambrosio R, Dentice M (2017). Thyroid hormone promotes differentiation of colon cancer stem cells. Mol Cell Endocrinol.

[62] Brubaker PL (2018). Glucagon-like Peptide-2 and the regulation of intestinal growth and function. Compr Physiol.

[63] Lee J, Koehler J, Yusta B (2017). Enteroendocrine-derived glucagon-like peptide-2 controls intestinal amino acid transport. Mol Metab.

[64] Mutanen A, Pakarinen MP (2017). Serum fasting GLP-1 and GLP-2 associate with intestinal adaptation in pediatric onset intestinal failure. Clin Nutr.

[65] Sugimoto S, Kobayashi E, Fujii M (2021). An organoid-based organ-repurposing approach to treat short bowel syndrome. Nature.

[66] Norona J, Apostolova P, Schmidt D (2020). Glucagon-like peptide 2 for intestinal stem cell and Paneth cell repair during graft-versus-host disease in mice and humans. Blood.

[67] Chen ME, Naeini SM, Srikrishnaraj A (2022). Glucagon-like peptide-2 stimulates S-phase entry of intestinal Lgr5+ stem cells. Cell Mol Gastroenterol Hepatol.

[68] Smither BR, Pang HY, Brubaker PL (2016). Glucagon-like Peptide-2 requires a full complement of Bmi-1 for its proliferative effects in the murine small intestine. Endocrinology.

[69] Jeppesen PB, Gabe SM, Seidner DL (2018). Factors associated with response to teduglutide in patients with short-bowel syndrome and intestinal failure. Gastroenterology.

[70] Reisch N, Taylor AE, Nogueira EF (2019). Alternative pathway androgen biosynthesis and human fetal female virilization. Proc Natl Acad Sci U S A.

[71] Schiffer L, Arlt W, Storbeck KH (2018). Intracrine androgen biosynthesis, metabolism and action revisited. Mol Cell Endocrinol.

[72] Yu X, Li S, Xu Y (2020). Androgen maintains intestinal homeostasis by inhibiting BMP signaling via intestinal stromal cells. Stem Cell Reports.

[73] Tokarz VL, Macdonald PE, Klip A (2018). The cell biology of systemic insulin function. J Cell Biol.

[74] Tabibzadeh S (2021). From genoprotection to rejuvenation. Front Biosci (Landmark Ed).

[75] Zhou W, Rowitz BM, Dailey MJ (2018). Insulin/IGF-1 enhances intestinal epithelial crypt proliferation through PI3K/Akt, and not ERK signaling in obese humans. Exp Biol Med (Maywood).

[76] Strilbytska OM, Semaniuk UV, Storey KB (2020). Insulin signaling in intestinal stem and progenitor cells as an important determinant of physiological and metabolic traits in drosophila. Cells.

[77] Zhang Y, Chua S (2017). Leptin function and regulation. Compr Physiol.

[78] Matsumura S, Kurashima Y, Murasaki S (2020). Stratified layer analysis reveals intrinsic leptin stimulates cryptal mesenchymal cells for controlling mucosal inflammation. Sci Rep.

[79] Bartucci M, Svensson S, Ricci-Vitiani L (2010). Obesity hormone leptin induces growth and interferes with the cytotoxic effects of 5-fluorouracil in colorectal tumor stem cells. Endocr Relat Cancer.

[80] Nicholls AR, Holt RI (2016). Growth hormone and insulin-like growth factor-1. Front Horm Res.

[81] Chen Y, Tsai YH, Tseng BJ (2019). Influence of growth hormone and glutamine on intestinal stem cells: a narrative review. Nutrients.

[82] Ulshen MH, Dowling RH, Fuller CR (1993). Enhanced growth of small bowel in transgenic mice overexpressing bovine growth hormone. Gastroenterology.

[83] Peng H, Poovaiah N, Forrester M (2015). Ex vivo culture of primary intestinal stem cells in collagen gels and foams. ACS Biomater Sci Eng.

[84] Bühler C, Hammon H, Rossi GL (1998). Small intestinal morphology in eight-day-old calves fed colostrum for different durations or only milk replacer and treated with long-R3-insulin-like growth factor I and growth hormone. J Anim Sci.

[85] Chen Y, Tseng SH, Yao CL (2018). Distinct effects of growth hormone and glutamine on activation of intestinal stem cells. JPEN J Parenter Enteral Nutr.

[86] Ampofo E, Nalbach L, Menger MD (2020). Regulatory mechanisms of somatostatin expression. Int J Mol Sci.

[87] Modarai SR, Opdenaker LM, Viswanathan V (2016). Somatostatin signaling via SSTR1 contributes to the quiescence of colon cancer stem cells. BMC Cancer.

[88] Makrigiannakis A, Vrekoussis T, Zoumakis E (2018). CRH receptors in human reproduction. Curr Mol Pharmacol.

[89] Miehlke S, Verhaegh B, Tontini GE (2019). Microscopic colitis: pathophysiology and clinical management. Lancet Gastroenterol Hepatol.

[90] Xu YJ, Sheng H, Wu TW (2018). CRH/CRHR1 mediates prenatal synthetic glucocorticoid programming of depression-like behavior across 2 generations. Faseb J.

[91] Li B, Lee C, Filler T (2017). Inhibition of corticotropin-releasing hormone receptor 1 and activation of receptor 2 protect against colonic injury and promote epithelium repair. Sci Rep.

[92] Cipolla-Neto J, Amaral FGD (2018). Melatonin as a hormone: new physiological and clinical insights. Endocr Rev.

[93] Lee JH, Yun CW, Han YS (2018). Melatonin and 5-fluorouracil co-suppress colon cancer stem cells by regulating cellular prion protein-Oct4 axis. J Pineal Res.

[94] Li L, Yin X, Meng H (2020). Increased progastrin-releasing peptide expression is associated with progression in gastric cancer patients. Yonsei Med J.

[95] Giraud J, Failla LM, Pascussi JM (2016). Autocrine secretion of progastrin promotes the survival and self-renewal of colon cancer stem-like cells. Cancer Res.

[96] Jin G, Sakitani K, Wang H (2017). The G-protein coupled receptor 56, expressed in colonic stem and cancer cells, binds progastrin to promote proliferation and carcinogenesis. Oncotarget.

[97] Giraud J, Foroutan M, Boubaker-Vitre J (2021). Progastrin production transitions from Bmi1(+)/Prox1(+) to Lgr5(high) cells during early intestinal tumorigenesis. Transl Oncol.

